# Atherogenic index of plasma is an independent predictor of mitral annular calcification

**DOI:** 10.1186/s12872-022-02891-4

**Published:** 2022-11-30

**Authors:** Sezen Baglan Uzunget, Kader Eliz Sahin

**Affiliations:** 1 Department of Cardiology, Sincan State Hospital Ankara, Ankara, Turkey; 2 Department of Cardiology, Adiyaman University Education and Research Hospital, Adiyaman, Turkey

**Keywords:** Atherogenic indices, Mitral annular calcification, Atherogenic index of plasma

## Abstract

**Background:**

In the latest reports, atherogenic indices have been related to acute coronary syndromes, stable coronary artery disease, heart failure and future cardiac events. Conventional atherosclerosis risk factors have been associated with mitral annular calcification (MAC), but data on the relationship between atherogenic indices and MAC are lacking. We aimed to investigate a possible relationship between MAC and atherogenic indices.

**Methods:**

In total 741 patients (n = 427 with MAC and n = 314 without MAC) who were examined in our cardiology clinic from February 2016 to October 2021 were recruited in the study. Mitral annular calcification was diagnosed by transthoracic 2-dimensional echocardiography. The atherogenic coefficient (AC), Castelli risk index 1 (CRI-1), Castelli risk index 2 (CRI-2) and atherogenic index of plasma (AIP) were calculated by utilizing standard lipid test values.

**Results:**

There was no statistically significant difference in sex, age, diabetes and hypertension status between the patient and the control groups. Serum triglyceride level, AIP, Hs-CRP, smoking and BMI were independently significantly associated with MAC in multiple regression analysis (p < 0.001).

**Conclusion:**

Higher AIP was related to the existence of MAC and also predict the presence of MAC independently. Studies evaluating the modification of these indices are needed.

**Supplementary information:**

The online version contains supplementary material available at 10.1186/s12872-022-02891-4.

## Introduction

Mitral annular calcification (MAC) is described as calcification of the fibrous structures of the mitral valve [1]. This chronic and degenerative process of the mitral valve is associated with cardiovascular diseases (CVD) and their risk factors [2–7]. Based on previous studies, it can be considered that atherosclerosis and MAC have mutual pathophysiology [8, 9]. However, the pathogenesis of MAC has not been fully elucidated and a definitive medical treatment has not yet been defined. Besides, understanding the etiopathogenesis and subsequent treatment strategies, MAC has become notable once again in recent years due to related intra- and postoperative complications during percutaneous valve implantations.

Atherogenic dyslipidemia, which is involved in the pathological process of atherosclerosis, is defined as coexistence of increased circulating triglycerides (TG) and decreased high-density lipoprotein cholesterol (HDL-C) [10]. While elevated triglycerides impair endothelial function by producing proinflammatory mediators, coagulation factors, and fibrinogen [11]. HDL-C has protective effects on endothelium by counteracting the migration of macrophages and increasing the outflow of oxidized cholesterol from these cells [12]. Previous studies have shown that atherogenic dyslipidemia, high total cholesterol (TC), circulating TG and lipoprotein (a) level were related to MAC [7, 13].

The atherogenic index of plasma (AIP), calculated according to log10 (TG/HDL-C) formula, has been proposed as a strong marker in prediction of the atherogenicity and cardiovascular events in addition to individual lipid risk parameters [14–18]. Compared to simple lipid parameters, AIP has more powerful associations with CVD and superior predictive ability for cardiovascular events [14]. However, it is unknown whether AIP values are associated with MAC. As AIP and the cholesterol derived other indices - the atherogenic coefficient (AC), Castelli risk index 1 (CRI-1), Castelli risk index 2 (CRI-2)- are all associated with CVD risk, we aimed in the present study to evaluate the relationship between AIP, AC, CRI-1, CRI-2 levels and MAC.

## Patients and methods

### Patient selection

**Fig. 1:**
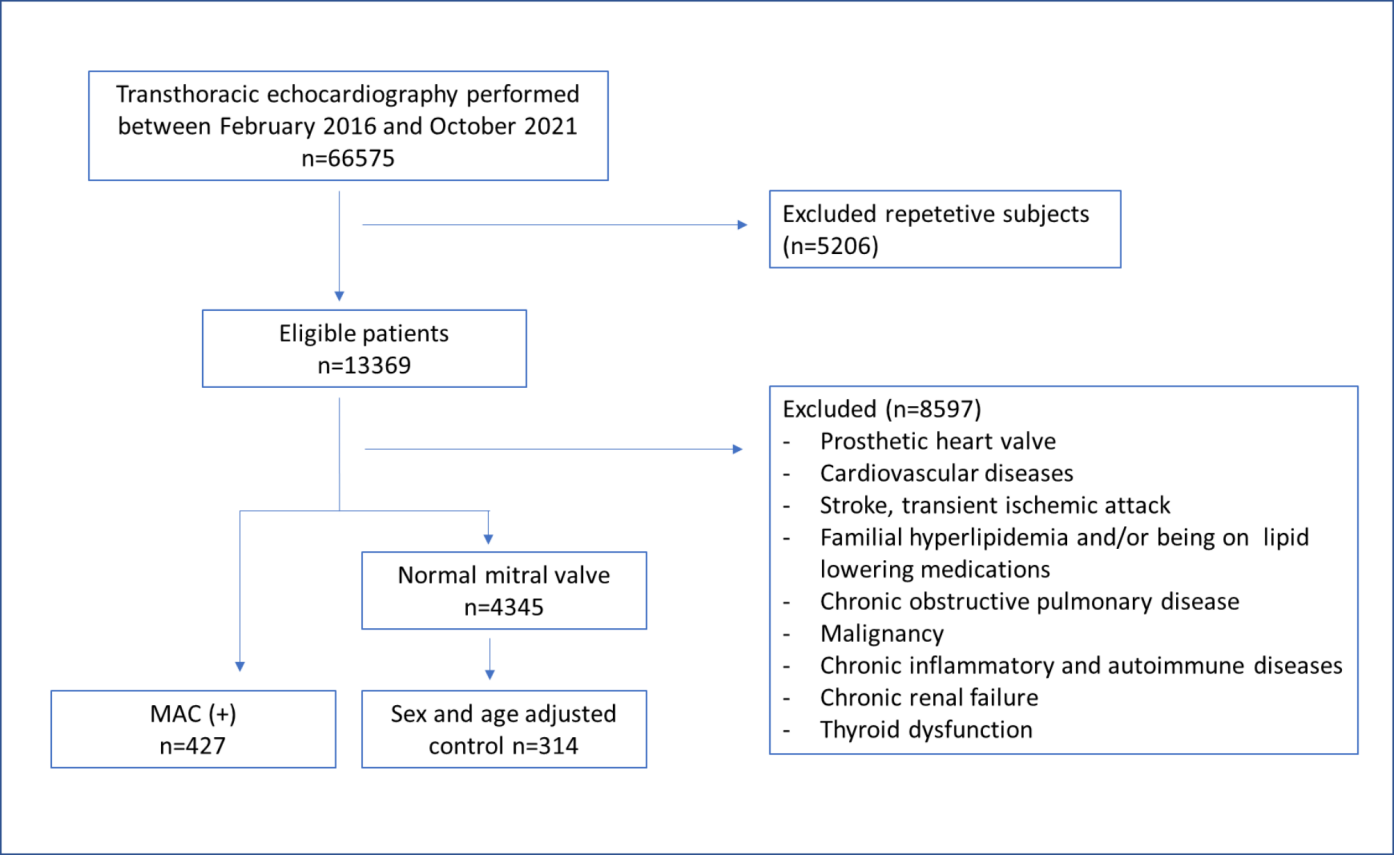
Flow chart of patient selection for the study

In the retrospective study, we scrutinized the data of 66,575 patients who had transthoracic echocardiography (TTE) between February 2016 and October 2021 at our outpatient clinic. The patients were chosen arbitrarily from our clinic TTE database and patient files. We excluded the patients who had missing data on admission. After applying the exclusion criteria, 427 patients with MAC and sex and age-adjusted 314 patients without MAC as control were enrolled in the study (Fig. 1). The study was organized considering the Helsinki Declaration.

Patients with coronary artery disease, coronary revascularization (CABG, angioplasty or stenting), myocardial infarction, more than 50% occlusion of the carotid arteries, carotid artery surgery and/or stenting, history of valve surgery, decompensated heart failure, familial lipid metabolism disorders and patients who were receiving any lipid lowering medications (statins, fenofibrate, ezetimibe, bile acid sequestrants, nicotinic acid and gemfibrozil) were excluded. In addition, we defined stroke, transient ischemic attack, peripheral arterial disease (occlusive disease of low extremity arteries with angiographic or Doppler ultrasound evidence or with history of revascularization or amputation), chronic obstructive pulmonary disease, malignancy, chronic inflammatory disease, autoimmune disease, chronic renal failure, thyroid dysfunction as exclusion criteria.

Blood pressure was measured after 5 min rest in the morning and the mean value of two separate measurements was recorded. Hypertension was defined when the SBP was higher than 140 mmHg and/or DBP was higher than 90 mmHg or was on antihypertensive therapy. Diabetes mellitus was defined if the fasting blood glucose level was exceeding 126 mg/dl or receiving any diet or medications to reduce blood glucose. Patients were assigned as current smokers who continued smoking currently or ex-smoker who have quit smoking in the last six months. Body mass index was calculated using the formula; weight (kilogram)/square of height (metre [2].

### Echocardiography

Standardized echocardiograms were performed between February 2016 and October 2021 at our echocardiography laboratory. MAC was determined as presence of marked echo density at any of those; the atrioventricular groove, posterior and/or anterior valve and/or extending sub-valvular apparatus notwithstanding the degree of the calcification.

### Laboratory

Laboratory results were gathered from the hospital database. Blood samples were drawn in the morning after following minimum 8 h fasting. Plasma TC, HDL-C, low density lipoprotein cholesterol (LDL-C) and TG values were tested by standard laboratory methods. LDL-C was measured directly by LDL-C tests. AIP was calculated following the formula; logarithm of the TG to HDL-C ratio. Rest of the indices investigated in this study were calculated as; CRI-1 = TC/HDL-C; CRI-2 = LDL-C/HDL-C and AC non-HDL-C/HDL-C.

### Statistical analysis

Data were analyzed using SPSS 22.0 for Windows. Normality distribution of variables was assessed by using Kolmogorov Smirnov//Shapiro-Wilk test. For comparison, Student t test, chi-square test, and Mann-Whitney U test were used as appropriate. Results were reported as mean ± standard deviation for continuous variables which were normally distributed, median (min-max) for continuous variables without normal distribution and percentages for qualitative variables. The receiver operating characteristic curve analysis was used to determine predictive values of risk factors for MAC. We performed multivariate regression analysis adjusted for the variables that were of statistical significance in the univariate analysis. A multivariable logistic regression model with backward selection (P > 0.1 as exit criterion) was used to evaluate variables associated with MAC. Odd’s ratio and 95% confidence intervals for each variable were calculated. The statistical significance level was accepted as p-value < 0.05 in all analyses.

## Results

We included 427 MAC (+) patients and 314 control subjects. The clinical, laboratory and demographic features of the patients are summarized in Table 1. There were no statistically significant difference in age, sex, diabetes and hypertension status between the patient and the control groups. The mean body mass index (BMI) and the incidence of smoking were significantly higher in the MAC (+) group (p < 0.001). TC, TG and LDL-C levels were significantly higher in the MAC group while HDL-C levels were significantly lower compared to the control (p < 0.001). High-sensitivity C-reactive protein (Hs-CRP), PAI, AC, CRI-1 and CRI-2 values were also significantly higher in the MAC group (p < 0.001).

**Table 1:** Demographic, clinical and laboratory characteristics of patients

	MAC (+) n = 427	Control n = 314	P value
**Demographic and clinical parameters**			
Age (years)	65.2 ± 7.9	65.8 ± 8.7	0.389*
Female, n (%)	256 (60.0%)	186 (59.2%)	844^†^
BMI (kg/m^2)^	26.8 ± 2.2	25.1 ± 2.5	< 0.001*
Smoking, n (%)			< 0.001^†^
No Current smoker Ex-smoker	233 (54.6%)	212 (67.5%)	
	128 (30.0%)	92 (29.3%)	
	66 (15.5%)	10 (3.2%)	
Hypertension, n (%)	196 (45.9%)	138 (43.9%)	0.598^†^
Diabetes mellitus, n (%)	215 (50.4%)	135 (43.0%)	0.047^†^
**Laboratory parameters**			
White blood cell (10^3^/µL)	6.8 ± 1.3	6.5 ± 1.7	0.025*
Hemoglobin (g/L)	13.5 ± 1.0	13.6 ± 1.2	0.255*
Platelet (10^3^/µL)	247.0 ± 41.1	251.7 ± 51.8	0.166*
Total cholesterol (mg/dL)	208.5 ± 28.6	200.0 ± 34.2	< 0.001*
HDL-C (mg/dL)	40.8 ± 7.2	47.3 ± 10.0	< 0.001*
LDL-C (mg/dL)	130.1 ± 24.3	124.7 ± 28.5	0.005*
Triglyceride (mg/dL)	187.5 ± 62.3	139.9 ± 61.1	< 0.001*
GFR (ml/dk)	86.2 ± 14.9	86.7 ± 11.9	0.639*
Hs-CRP (mg/L)	3.50 ± 2.5	2.28 ± 2.0	< 0.001*
Atherogenic index of plasma	0.64 ± 0.18	0.44 ± 0.20	< 0.001*
Atherogenic coefficient	4.25 ± 1.11	3.39 ± 1.09	< 0.001*
Castelli’s risk index 1	5.25 ± 1.11	4.39 ± 1.09	< 0.001*
Castelli’s risk index 2	3.29 ± 0.91	2.77 ± 0.92	< 0.001*

Atherogenic coefficient (non-HDLc/HDLc), Atherogenic index of plasma (log triglyceride/HDL-C), *BMI* body mass index; Castelli’s risk index 1(total cholesterol/HDLc), Castelli’s risk index 2 (LDLc/HDLc), *GFR* glomerular filtration rate, *Hs-CRP* high-sensitive C-reactive protein, *HDL-C* high density lipoprotein cholesterol, *LDL-C* low density lipoprotein cholesterol

* Mean ± SD in cases where the data were distributed normally, analyzed by student’s t test

^†^ analyzed by qi-square test

When we compared echocardiographic parameters (Table 2), left ventricular end-diastolic diameter (LVEDD), left ventricular end-systolic diameter (LVESD), left atrium diameter (LAD) and systolic pulmonary arterial pressure (sPAP) of the MAC (+) patients were significantly higher than the control (p < 0.001). There were no significant statistical difference among mitral stenosis, mitral regurgitation and aortic regurgitation frequencies between the groups (p = 0.103, p = 0.264 and p = 0.417 respectively), while aortic sclerosis and mild to moderate aortic stenosis rates were higher in the MAC (+) group (p = 0.046 and p = 0.001).

**Table 2:** Echocardiographic comparison of patients

	MAC (+) n = 427	Control n = 314	P value
LVEF (%)	57.8 ± 8.4	58.8 ± 7.3	0.103*
LVEDD (cm)	4.8 ± 0.3	4.7 ± 0.3	< 0.001*
LVESD (cm)	3.3 ± 0.4	3.2 ± 0.4	< 0.001*
IVSd (cm)	0.97 ± 0.1	0.99 ± 0.1	0.023*
PWd (cm)	0.97 ± 0.1	0.97 ± 0.1	0.293*
LAD (cm)	4.1 ± 0.6	3.7 ± 0.5	< 0.001*
sPAP (mmHg)	34.3 ± 6.4	29.9 ± 5.9	< 0.001*
Mitral stenosis, n (%)			0.103^†^
No	393 (92.0%)	301 (95.9%)	
Mild	30 (7.2%)	11 (3.5%)	
Moderate	4 (0.8%)	2 (0.6%)	
Mitral regurgitation, n (%)			0.264^†^
No	308 (72.1%)	243 (77.4%)	
Mild	101 (23.7%)	61 (19.4%)	
Moderate	18 (4.2%)	10 (3.2%)	
Aortic sclerosis, n (%)	126 (29.5%)	72 (22.9%)	0.046^†^
Aortic stenosis, n (%)			0.001^†^
No	348 (81.5%)	289 (92.0%)	
Mild	52 (12.2%)	19 (6.1%)	
Moderate	25 (5.9%)	6 (1.9%)	
Severe	2 (0.4%)	0	
Aortic regurgitation, n (%)			0.417^†^
No	366 (85.7%)	276 (87.9%)	
Mild	52 (12.2%)	35 (11.1%)	
Moderate	9 (2.1%)	3 (1.0%)	

*IVSd* interventricular septum thickness, *LAD* left atrium diameter, *LVEDD* left ventricular end-diastolic diameter, *LVESD* left ventricular end-systolic diameter, *LVEF* left ventricular ejection fraction, *PWd* posterior wall thickness, *sPAP* systolic pulmonary arterial pressure.

*Mean ± SD in cases where the data were distributed normally, analyzed by student’s t test

^†^analyzed by qi-square test

**Fig. 2:**
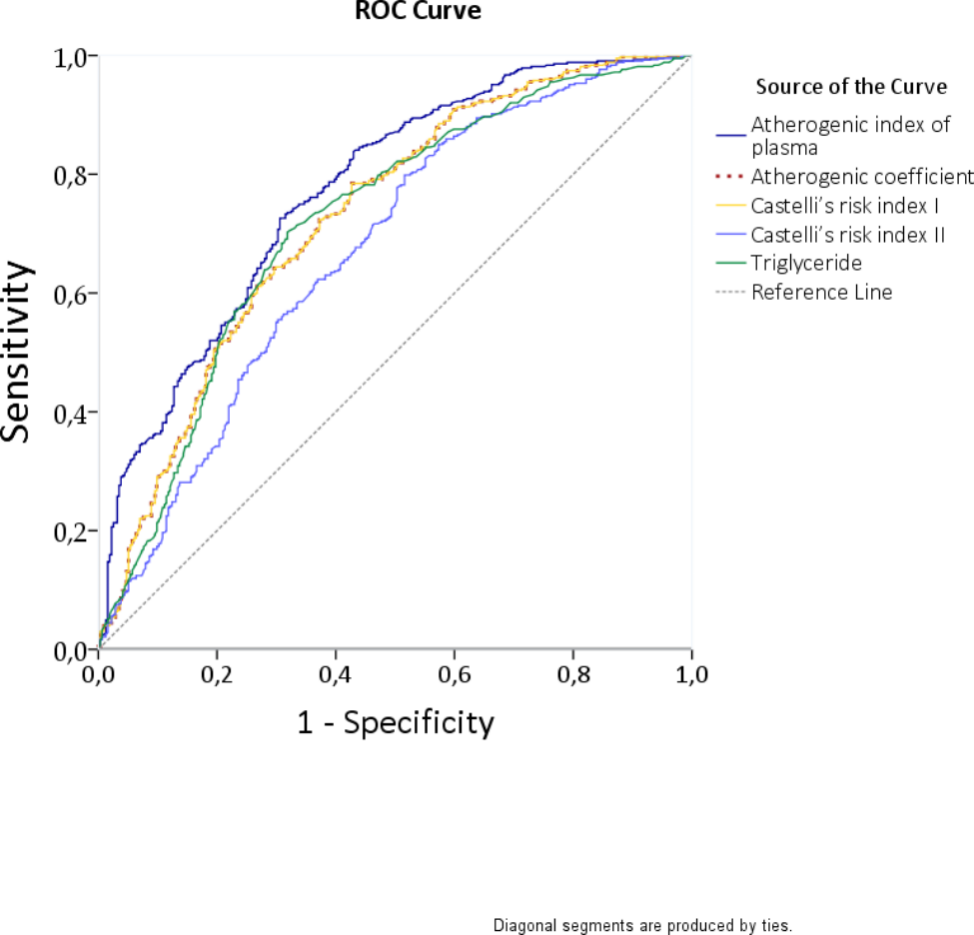
The receiver operating characteristic curve for predictive value of risk factors for MAC.

ROC curve analysis revealed a cut-off level of 0.56 for AIP to predict MAC with 71% sensitivity and 70% specificity (AUC: 0.772, p < 0.001, 95% CI: 0.738–0.806) (Fig. 2; Table 3).

**Table 3:** Area under curve table for risk factors associated with mitral annular calcification

Area Under the Curve								
Test Result Variable(s)	Area	Std. Error^a^	Cut-off point	Sensitivity (%)	Specificity (%)	P^b^	Asymptotic 95% Confidence Interval	
							Lower Bound	Upper Bound
Atherogenic index of plasma	0.772	0.017	0.56	71	70	< 0.001	0.738	0.806
Atherogenic coefficient	0.728	0.019	3.63	68	65	< 0.001	0.690	0.765
Castelli’s risk index 1	0.728	0.019	4.63	68	65	< 0.001	0.690	0.765
Castelli’s risk index 2	0.672	0.020	2.93	64	62	< 0.001	0.632	0.712
Triglyceride	0.719	0.019	150.5	68	69	< 0.001	0.681	0.757

The test result variable(s): Atherogenic index of plasma, Atherogenic coefficient, Castelli’s risk index 1, Castelli’s risk index 2, Triglyceride has at least one tie between the positive actual state group and the negative actual state group. Statistics may be biased

^a^Under the nonparametric assumption

^b^Null hypothesis: true area = 0.5

The associations of possible risk factors for MAC were assessed by univariate and multivariate logistic regression analyses (Table 4). Among all lipid parameters and lipid derived indices, only serum TG level and AIP were independently and significantly associated with MAC in multiple regressions (OR: 0.988; 95% CI: 0.982–0.994 and OR: 2.460; 95% CI: 1.984–3.049, p < 0.001). Hs-CRP, smoking and BMI were also independently and significantly associated with MAC presence (OR: 1.250; 95% CI: 1.150–1.359, OR: 2.129; 95% CI: 1.584–2.862, OR: 1.253; 95% CI: 1.159–1.356 respectively, p < 0.001).

**Table 4:** Possible risk factors for mitral annular calcification in univariate and multivariate logistic regression analyses

	Univariate analysis		Multivariate analysis	
	OR (95% CI)	***P***	OR (95% CI)	***P***
Age	0.992 (0.975–1.010)	0.389		
Sex	0.971 (0.721–1.306)	0.844		
BMI	1.342 (1.252–1.439)	< 0.001	1.256 (1.162–1.358)	< 0.001
Smoking	1.805 (1.428–2.282)	< 0.001	1.954 (1.344–2.839)	< 0.001
DM	1.345 (1.003–1.803)	0.048	1.353 (0.938–1.951)	0.105
HT	1.082 (0.807–1.451)	0.598		
HL	2.629 (1.942–3.558)	< 0.001	1.369 (0.919–2.041)	0.123
Hs-CRP	1.272 (1.182–1.370)	< 0.001	1.241 (1.143–1.348)	< 0.001
WBC	1.117 (1.014–1.231)	0.026	1.057 (0.933–1.197)	0.386
GFR	0.997 (0.987–1.008)	0.639		
Total cholesterol	1.009 (1.004–1.014)	< 0.001		
Triglyceride	1.012 (1.010–1.015)	< 0.001	0.984 (0.976–0.991)	< 0.001
LDL	1.008 (1.002–1.014)	0.006	1.004 (0.989–1.019)	0.597
HDL	0.914 (0.896–0.932)	< 0.001		
AIP	1.751 (1.591–1.925)	< 0.001	2.431 (1.964–3.008)	< 0.001
AC	2.186 (1.848–2.585)	< 0.001	0.841 (0.641–1.104)	0.213
CRI 1	2.186 (1.848–2.585)	< 0.001		
CRI 2	1.959 (1.629–2.357)	< 0.001	1.340 (0.039–46.221)	0.871

*AC* atherogenic coefficient (non-HDLc/HDLc), *AIP* atherogenic index of plasma (log triglyceride/HDL-C), *BMI* body mass index, *CRI 1* Castelli’s risk index 1 (total cholesterol/HDLc), *CRI 2* Castelli’s risk index 2(LDLc/HDLc), *DM* diabetes mellitus, *Hs-CRP* high-sensitive C-reactive protein, *WBC* white blood cell.

## Discussion

In the present study, we compared conventional lipid parameters, AIP, AC, CRI-1 and CRI-2 in patients with MAC and without MAC. We demonstrated that the AIP, Hs-CRP, smoking and BMI were significantly associated with the presence of MAC independent of other baseline clinical risk factors by the multivariate analysis.

Mitral annular calcification is characterized by calcium accumulation along the mitral annular ring, more specifically its posterior side, and affects the anterior part of the structure with the severity of the pathology [2]. In an earlier autopsy series, examination of mitral annulus samples revealed that the lipid and calcium deposition in the collagen tissue increased with the age [19]. Although in older studies, it was considered an age-related deformation of the valve structure, recent reports have demonstrated that it is an active inflammatory process that accompanies lipid displacement and bone formation [20]. Despite its high prevalence in general population ranging from 5 to 42%, the pathophysiology of MAC is not fully understood [2]. Since effective medical treatment is still lacking and percutaneous and surgical interventions are complicated by the presence of MAC, we think that it is worth working on the subject in order to elucidate the mechanism and related pathologies and to base it on the development of treatment options.

Although its mechanism is not fully resolved, one possibility is that the atrioventricular groove, in which blood flow turbulence increases and shear stress decreases, may constitute a medium for lipid and calcium deposition. Previous studies showed that the endothelium damage and dysfunction (as the first step) and ongoing inflammation accompanying lipid accumulation were the main pathological mechanisms of MAC [20–22]. Also, it is accepted that, foam cells formed by macrophages that engulfed LDL-C particles after lipid accumulation in the mitral annulus, are not supported metabolically sufficiently and they turn into calcium deposits as a result of apoptosis [2, 8, 23]. Apart from low shear rate and increased blood flow turbulence, high circulating atherogenic lipids which are defined as elevated, serum triglycerides, apolipoprotein B and small dense low-density lipoprotein cholesterol (sdLDL-C) accompanying low HDL-C levels, are implicated in the genesis of this process [24, 25]. In contrast to high LDL-C and triglyceride levels, HDL-C has a protective effect on this pathology [12]. Oxidized LDL impedes microcirculation by inhibiting nitric oxide synthase which is regulated in the endothelium and thus conduces to the atherosclerotic plaque development [26]. HDL saves endothelial cells from the detrimental activity of LDL-C and ameliorates endothelial cell dysfunction [12]. On the other hand, it has been shown that hypertriglyceridemia activates the inflammatory mediators and coagulation cascade, leading to endothelial dysfunction [11, 27]. However, as a subfraction of LDL-C molecule, sdLDL-C value is considered to be more accurate and stronger indicator of atherogenesis [28–30]. In contrast to this success, sdLDL-C measurement is not a cost-effective method in clinical settings [7, 29–31]. Nonetheless, AIP derived from routine lipid parameters has been accepted to reflect sdLDL-C particle measurement [31].

As a prominent index of atherogenicity in recent publications, AIP that is formulated as log10 (TG/HDL-C) showed higher correlations with cardiovascular diseases and stronger predictive power for future cardiovascular outcome when compared to simple lipid measurements in previous studies [16, 17]. As emerging data increasingly endorse potential relation with atherogenic indices and endothelial dysfunction and subclinical atherosclerosis, we focused on the link between atherogenic indices and MAC to contribute to the data in the literature. We have demonstrated a positive correlation between AIP and presence of MAC in this study. This relationship between MAC and AIP supports the view that MAC and atherosclerosis have common risk indicators likewise obesity, hypertension, dyslipidemia and diabetes mellitus [32]. Furthermore, AC, CRI-1 and CRI-2 were higher in the MAC group than in the control group. However, these parameters lost their predictive value in the regression analysis.

In the study of Allison et al., patients with MAC have had higher non-HDL-C levels, lower HDL-C levels and higher TC to HDL-C ratios than age and sex matched controls while there was no significant difference in TC levels [33]. Nonetheless, Afshar et al. reported that elevated triglyceride levels in MAC patients were significant attributing that the principal reason of the pathology was hidden in the genes [13]. They reported that only triglyceride genetic risk scores were associated with MAC but not LDL-C and HDL-C genetic risk scores. Besides, in a prospective trial by O’Neal et al., there were no differences in TC and HDL-C levels in MAC positive and negative patients which may be due to that they did not exclude patients receiving lipid lowering medications in contrast to our study [5]. In addition to genetic predisposition, the widely proven association between coronary artery disease, aortic valve calcification, MAC and atherogenic dyslipidemia and even the success of lipid-lowering therapy in plaque regression cannot be denied whatever the causes. Although the lipid lowering treatment failed to heal the aortic valve calcification, the impact of this therapy on MAC remains unrevealed [34, 35]. Considering that the calcifications seen in the mitral annulus and aortic valve are the results of idiosyncratic mechanisms, lipid-lowering therapy in MAC should be contemplated and studied separately [36].

We have reported that increased smoking status, BMI and Hs-CRP levels were independently associated with MAC like some previous studies [37]. Although, we found a significant relationship between Hs-CRP levels and MAC, the role of inflammation is still debatable in the disease. In a study examining the Framingham offspring cohort, CRP values were significantly higher in participants with MAC, but lost its significance after adjusting for other cardiovascular risk factors [38]. Unlike this study, we analyzed Hs-CRP measurements with higher sensitivity and CRP measurements could not reflect the local tissue inflammation as much as Hs-CRP despite larger population of their study. In another large-scale study involving 6464 participants, glycoprotein acetylation (GlycA), which is an indicator of cumulative inflammatory response, was found to be associated with the incidence of MAC after adjusting age, sex, BMI, and smoking even predicted MAC progression in the tomographic evaluation during the 2-year follow-up [39].

## Limitation

Our study has some restrictions. Firstly, current study was designed retrospectively which emerged a structural limitation. Secondly, we did not evaluate the severity of MAC by echocardiography or any other imaging modalities. Thirdly, the echocardiography evaluation may have limitations due to imaging quality of echocardiography imaging method itself, but all the patients tested by the same device. Next, hypertension was diagnosed after two separate measurements of blood pressure on one visit though 2–3 office visits at 1–4-week intervals are suggested to confirm the diagnosis of hypertension. Lastly, our analysis based on a spot blood measurement so the alterations of those parameters during long periods could not be assessed meaning that lack of prognostic role in those patients.

## Conclusion

Elevated atherogenic indices have been associated with the existence of MAC, which has an increased risk for mitral valvular disease, conduction system delays and atrial fibrillation. Future investigations are warranted to elucidate whether lipid lowering medication could prevent the mitral annular calcification.

## Electronic supplementary material

Below is the link to the electronic supplementary material.

Supplementary Material 1: Mac raw data

Supplementary Material 2: Supplement file for ROC curve analysis

Supplementary Material 3: Legends of supplementary file

## Data Availability

We added the raw data in the supplementary part of the journal’s web site. The dataset generated and/or analysed during the current study is not publicly available due to we do not have consents of patients to publish this data. However, it is available from the corresponding author on reasonable request.
