# Supplementary material for: Atherogenic index of plasma is an independent predictor of mitral annular calcification
Source: BMC Cardiovasc Disord. 2022 Nov 30;22:511. doi: 10.1186/s12872-022-02891-4 (PMC9710030; doi:10.1186/s12872-022-02891-4)
Supplement: Supplementary file 3 — Supplementary Material 3: Legends of supplementary file [file 12872_2022_2891_MOESM3_ESM.docx]

**Legend of supplementary file:**

1. Raw dataset of the study as mac raw data.xlsx

2. Detailed roc curve analysis
